# Lightning initiation: Strong pulses of VHF radiation accompany preliminary breakdown

**DOI:** 10.1038/s41598-018-21972-z

**Published:** 2018-02-26

**Authors:** Ivana Kolmašová, Ondřej Santolík, Eric Defer, William Rison, Sylvain Coquillat, Stéphane Pedeboy, Radek Lán, Luděk Uhlíř, Dominique Lambert, Jean-Pierre Pinty, Serge Prieur, Véronique Pont

**Affiliations:** 1 Institute of Atmospheric Physics, The Czech Academy of Sciences, Prague, Czechia; 2 Faculty of Mathematics and Physics, Charles University, Prague, Czechia; 3 Laboratoire d’Aérologie, Université de Toulouse, CNRS, OMP, UPS, Toulouse, France; 4 Electrical Engineering Department, New Mexico Tech, Socorro, New Mexico United States; 5 Météorage, Pau, France

## Abstract

We analyze lightning initiation process using magnetic field waveforms of preliminary breakdown (PB) pulses observed at time scales of a few tens of microseconds by a broad-band receiver. We compare these pulses with sources of narrow-band very high frequency (VHF) radiation at 60–66 MHz recorded by two separate Lightning Mapping Arrays (LMAs). We find that almost none of the observed PB pulses correspond to geo-located VHF radiation sources, in agreement with previous results and with the hypothesis that processes generating VHF radiation and PB pulses are only weakly related. However, our detailed analysis discovers that individual peaks of strong VHF radiation seen by separate LMA stations correspond surprisingly well to the PB pulses. This result shows that electromagnetic radiation generated during fast stepwise extension of developing lightning channels is spread over a large interval of frequencies. We also show that intense VHF radiation abruptly starts with the first PB pulse and that it is then continuously present during the entire PB phase of developing discharges.

## Introduction

Processes occurring inside the thundercloud and leading to lightning initiation have been studied for several decades^1–4^ and continue to be a subject of intense research^5–8^. These processes start by impulsive electrical currents emitting a sequence of bipolar electromagnetic pulses which are believed to indicate preliminary breakdown processes inside the cloud^9^. This sequence - called “preliminary breakdown” (PB) pulses throughout this study - is often observed at the beginning of the pulse activity preceding the first return stroke (RS) of the cloud-to-ground lightning or during the early stage of intracloud lightning. Each individual PB pulse is a few tens of microseconds wide and the largest PB pulses are typically bipolar with the initial polarity identical to the polarity of the following RS pulse^10^.

The peak-to-peak amplitudes of the largest PB pulses are usually smaller than the amplitude of the corresponding RS pulse but sometimes they can also be comparable^2,10,11^. The sequence of PB pulses is usually followed by a relatively low and irregular pulse activity lasting from several tens to a few hundreds of milliseconds before the onset of the stepped leader and RS^11^. However, in some cases the pre-stroke activity does not lead to a regular RS pulse and the preliminary breakdown pulse activity remains isolated^12–14^. The PB pulses, also known as initial breakdown (IB) pulses, were observed to occur during repeating stepwise extension of the in-cloud lightning channel^4^, but exact generation mechanisms of sequences of PB pulses are still unknown.

A recent study^15^ suggests that radio pulses observed during the lightning initiation are generated by synchronized micro-discharges at the tips of hydrometeors. The local additional ionization caused by micro-discharges then leads to an enhancement of local conductivity and to preconditioning of initial in-cloud lightning leaders. A similar mechanism has been identified for fast positive breakdown processes^16,17^ detected using high-speed very high frequency (VHF) interferometric measurements at 20–80 MHz. The fast positive breakdown process was observed to occur at the onset of a narrow bipolar event^16^, during in-cloud K-changes^17^, and/or following negative CG stroke^17^. It is believed to take place in the virgin air, and appears to indicate a streamer-like activity initiated by the corona from hydrometeors. A hypothesis that many or possibly all lightning flashes might be initiated by this fast positive breakdown process was supported by measurement of initiation of an upward negative intra-cloud leader^16^ (observed recently also by other interferometric measurements^18^). These conclusions were further supported by analysis of impulsive VHF radiation from a GPS based lightning mapping array^19,20^ (LMA) of narrow band receivers at 60 - 66 MHz. However, coincidence of geo-located VHF radiation sources from LMA with the measured electric field pulses was not investigated.

An initial small amplitude slowly developing change of the electric field was reported^21^ to precede the occurrence of the first PB pulse, starting a few hundreds of microseconds before it for a cloud-to-ground flash, and up to five milliseconds before it in the case of an intracloud flash. This initial change of the electric field was only detected when lightning occurred at short distances (within 7 km) of the electric field sensors sampling at 10 MHz. Most importantly, the authors found that the start of this change went along with an impulsive VHF radiation recorded and geo-located by the LMA at 60–66 MHz in 14 of 36 observed cases. In remaining cases impulsive VHF radiation occurred up to 5 ms after the first PB pulse. The time coincidence of geo-located VHF radiation sources with other PB pulses was not discussed, but the results indicate that these impulsive VHF radiation sources are not observed to occur at the same time as the individual PB pulses, implying that both initiation processes might be unrelated at scales of tens to hundreds of microseconds.

In another study^4^ the authors concluded that the bipolar electromagnetic PB pulses are usually not located by the VHF detection systems, implying again that impulsive VHF radiation sources are observed unrelated to the individual PB pulses. Several other studies^22–25^ used an LMA system of eight stations operating at 66–72 MHz in Florida, US with a capability to locate a VHF radiation source every 10 μs. Descending VHF radiation sources were observed during the initial breakdown stage of a negative cloud-to-ground flashe^23^ but the association of these VHF sources to individual PB pulses was not discussed. Initial breakdown periods of other analyzed flashes (8 cloud-to-ground and 7 intracloud flashes)^24^ always contained several geo-located VHF radiation sources which occurred unrelated to PB pulses, supporting thus the previously discussed absence of coincidence between the two phenomena.

This observation was further supported by other studies. For example, a statistical comparison of cosmic ray air shower data with the geo-located VHF radiation sources^25^ yielded negative results, with a hypothesis that initial breakdown of a lightning flash does not produce a significant VHF radiation. In another study^26^ the density of radiation sources was analyzed using a three-dimensional LMA system of narrow-band VHF receivers (267–273 MHz) and with electric-field measurements up to 10 MHz. The direction of propagation of a developing discharge has been obtained but time coincidences of the individual PB pulses and VHF radiation sources were not noticed.

These examples demonstrate that, based on existing literature, correspondence of sources of impulsive VHF radiation with PB pulses seems to be absent or very weak. That might mean that temporal and spatial scales of the underlying lightning initiation processes are very different for these two types of phenomena.

In the present study we experimentally investigate this hypothesis by analyzing the coincidence of magnetic-field pulses measured by a broad-band receiver (0.005–37 MHz, see Methods) with geo-located sources of impulsive VHF radiation and also with individual peaks of VHF radiation at separate LMA stations. We use narrow-band VHF data recorded at 60–66 MHz by two different LMA installations (see Methods for their description). These two groups of measurements were recorded at different places and different time periods by nearly identical sets of instrumentation.

## Result

**Figure 1:**
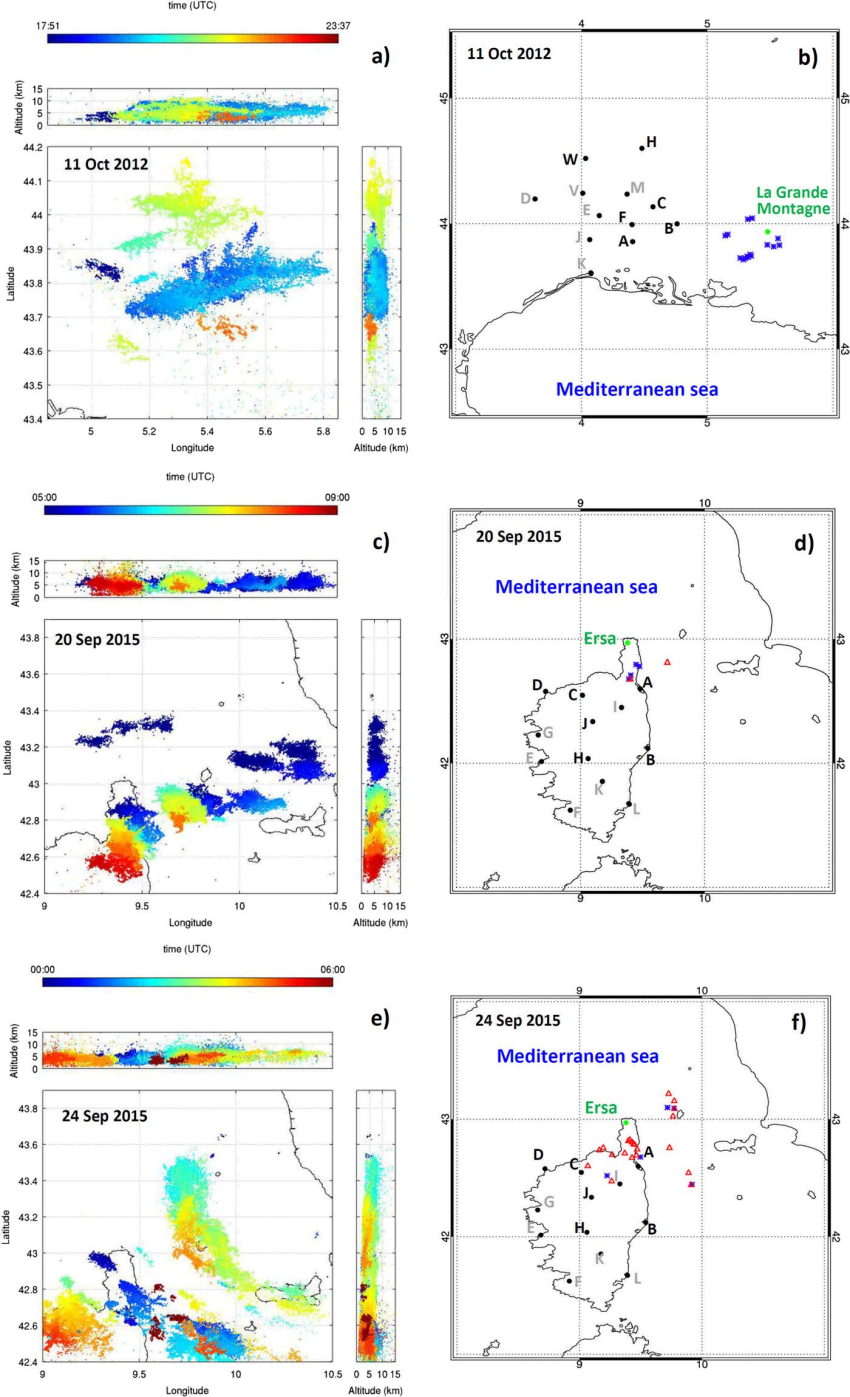
Maps. (**a**) VHF radiation sources geo-located by HyLMA on 11 October 2012. (**c,e**) VHF radiation sources geo-located by SAETTA on 20 September 2015 and 24 September 2015, respectively. Color represents the time of observation of each source. (**b**) Configuration of instruments used in 2012. (**d,f**) Configuration of instruments used in 2015. Green dots represent locations of broadband magnetic-field receivers, black dots show positions of LMA stations, blue stars and red triangles respectively represent negative cloud-to-ground and intracloud discharges identified by Météorage (no positive cloud-to-ground lightning was detected). The LMA stations used for our study are labeled by dark letters. Maps have been plotted using the Interactive Data Language ver. 8.6.0.

The first dataset of analyzed broad-band magnetic field measurements was collected during a thunderstorm which occurred on 11 October 2012 within 30 km from the measurement site La Grande Montagne (Fig. 1a,b). This dataset consists of 15 sequences of PB pulses which were followed by negative RS pulses. These measurements were already used^5,6^ for analysis of properties of unusually short pulse sequences occurring prior to the first strokes of negative cloud-to-ground lightning flashes. The second dataset was collected during two thunderstorms occurring on 20 and 24 September 2015 within 73 km from the measurement site Ersa, Corsica (Fig. 1c,d and Fig. 1e,f respectively) and consists of 37 sequences of PB pulses. Of them, seventeen sequences were followed by RS pulses, and 20 sequences were not followed by RS pulses within the 208-ms records of magnetic-field waveforms. We have selected only the PB pulses which are clearly grouped in sequences in order to exclude isolated narrow bipolar pulses from our analysis.

These datasets are compared with the lists of geo-located VHF sources determined by two different LMA installations. Each list of geo-located VHF sources contains information about their locations (latitude, longitude and altitude) and emission time. We also investigate lists of separate peaks of radiated VHF power detected at individual LMA stations which record the reception time and received power of the strongest VHF sample within each 80 µs LMA window. These records of raw measurements are usually only used to reconstruct the 3D geo-location of the VHF sources. They also give us the number of 40-ns VHF samples whose power at the receiver exceeded an automatically determined threshold within each LMA window.

Overviews of the geo-located VHF radiation sources from LMA during the three analyzed time intervals are respectively are shown in Fig. 1a,c and e. Locations of the magnetic-field measurement sites, locations of LMA stations, and the locations of all discharges identified by the Météorage lightning location network (see Methods) are shown on corresponding larger scale maps in Fig. 1b,d and f.

### Analysis of the 2012 dataset

The main properties of 15 recorded sequences of magnetic-field PB pulses belonging to the 2012 dataset^5^ are: All observed PB pulse sequences are followed by negative cloud-to-ground RS pulses. Peak-to-peak amplitudes of the largest PB pulses are smaller than peak-to-peak amplitudes of corresponding RS pulses in all cases; the mean value of the ratio of the peak-to-peak amplitudes of the largest PB pulse and the corresponding RS pulse is about 0.2. The mean value of the time separation between the first PB pulse and the corresponding RS pulse is 2.5 ms.

**Figure 2:**
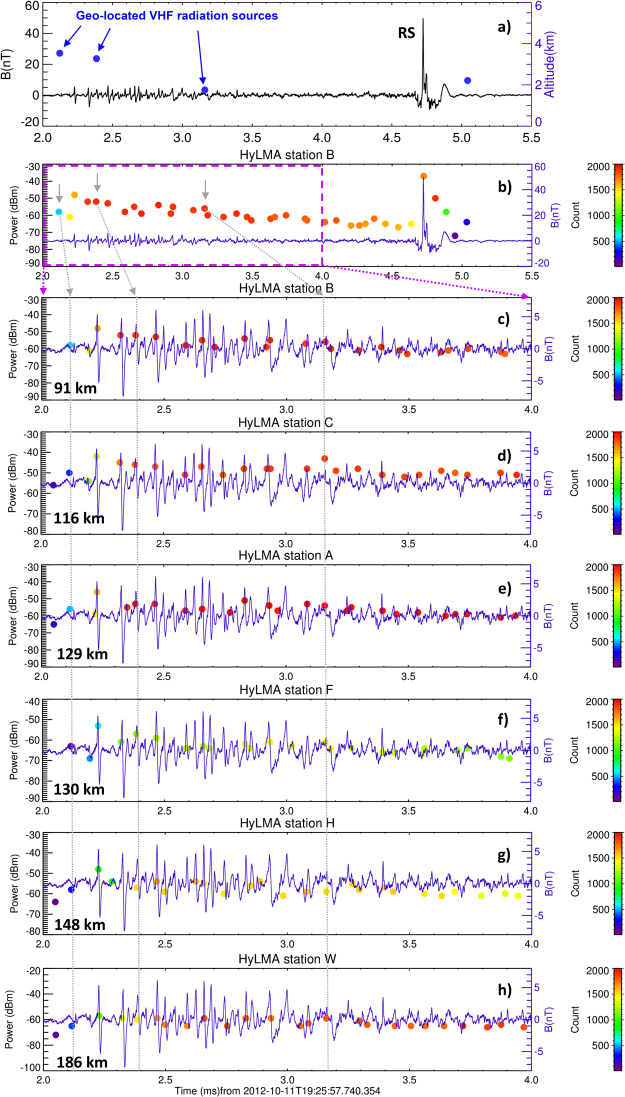
Example of a negative cloud-to-ground discharge from the 2012 dataset. A continuous broadband waveform recorded by BLESKA showsa sequence of PB pulses and the corresponding return stroke pulse (11 October 2012, 19:25:57.7 UTC, 5.30°E, 43.73°N) with (**a**), geo-located VHF radiation sources including their occurence altitudes from HyLMA (blue dots) and (**b**), peaks of radiated VHF power recorded at the closest HyLMA station B. The dots representing the occurrence of strongest VHF peaks are color-coded by the number of intense VHF samples recorded in the corresponding 80 µs LMA windows. The power of the strongest peak of radiated VHF power in the corresponding windows is shown on the left-hand vertical axis. (**c–h**) Plots show a 2-ms long detail of the same PB pulse sequence with the peaks of radiated VHF power detected at different HyLMA stations (B, C, A, F, H, and W). Gray arrows and lines identify the geo-located VHF radiation sources occurring within the pre-stroke period of the observed discharge with peaks of radiated VHF power recorded at individual stations. Propagation time between the discharges and the different sensors is taken into account to provide accurate temporal overlays. The expected accuracy of temporal overlays of broadband magnetic-field measurements and LMA detections is several µs but the LMA system reports only one (the strongest) peak of radiated VHF power in each 80 µs wide window.

The LMA was able to localize VHF radiation sources during the pre-stroke phase starting by the first PB pulse for 14 analyzed cases from the 2012 dataset. The number of geo-located sources during the pre-stroke phase of individual RS pulses varies from 0 to 6. The first geo-located source which can be linked to the analyzed PB sequences always appears within a 1 ms-wide window centered on the first detectable PB pulse. The height above the sea level of these geo-located VHF radiation sources varies from 2165 to 6007 m, corresponding approximately to the range of the initiation height of observed discharges inside the thundercloud. Only a small portion of the geo-located VHF radiation sources has been found to be clearly related to the PB pulses in any of the analyzed cases. Figure 2a illustrates an example of a broadband magnetic field waveform which includes the whole process starting by a sequence of PB pulses and ending by the corresponding RS pulse. The discharge occurred on 11 October 2012 at 19:25:57.7 UTC. Its location was reported by Météorage as 5.30°E, 43.73°N; its peak current reached −118 kA. Blue dots represent the geo-located VHF radiation sources, including their heights shown on the right-hand vertical axis. Their LMA locations are 1.36–2.23 km apart from the RS.

In order to examine the visibly poor temporal correspondence of broadband magnetic-field pulses and geo-located VHF radiation sources in Fig. 2a, we have also analyzed the raw data recorded by all 6 operating LMA stations (stations A, B, C, F, H, and W, shown by dark letters in Fig. 1b). The reception time of the broadband magnetic field waveform and of each peak of radiated VHF power was corrected to take into account the propagation delay. This correction assumes the speed of light propagation to the corresponding receiving station, based on the known location of a geo-located VHF radiation source occurring closest in time to the beginning of the sequence of PB pulses.

Having both the magnetic-field waveforms and the LMA signals synchronized, the time series of the VHF radiation peaks detected at each operating LMA station were overlaid on the magnetic field waveform. Figure 2b shows the same waveform as in Fig. 2a but with the peaks of radiated VHF power (colored dots) detected at the LMA station B, the closest one to the analyzed discharge. The dots are color-coded by the number of intense VHF samples (above the threshold) recorded at the LMA station B in the corresponding 80 µs LMA windows (see Methods). The power of the strongest peaks of radiated VHF power in these LMA windows is shown on the left-hand vertical axis. Figure 2c–h show peaks of radiated VHF power detected by all operating LMA stations, together with a 2-ms long temporal zoom of the sequence of PB pulses indicated by a magenta rectangle in Fig. 2b. The plots are sorted by the distance of a given LMA station to the discharge. The distances are indicated in each individual plot. As expected, counts of intense VHF samples within 80-µs time windows detected at closer LMA stations are very high, in many cases they are close to the maximum value of 2000. In other words, considering a sampling interval of 40 ns, the power of nearly all samples exceeded the threshold within the corresponding 80-µs time windows. This suggests rather continuous radiation within each of these windows. All 15 sequences from the 2012 datset have been analyzed in the same way and give similar results. The RS location was used to calculate the propagation correction in one case for which no VHF radiation source was geo-located in the time interval between the first PB pulse and the RS.

### Analysis of the 2015 dataset

The dataset from 2015 includes 37 sequences of PB pulses originating in two different thunderstorms. Both thunderstorms exhibited a substantially larger variability of the lightning initiation process in comparison with the dataset from 2012. The strongest PB pulses within the individual sequences were detected by Météorage in 23 cases as intra-cloud discharges. Their estimated peak currents vary from 6 to 52 kA, with a mean value of about 13 kA. The dataset contains 20 sequences which can be labeled as isolated breakdown pulses, not being followed by RS pulses within the 208-ms long magnetic field records.

The remaining 17 sequences of PB pulses are followed by RS pulses. The peak-to-peak amplitudes of the largest PB pulses are smaller than the peak-to-peak amplitudes of corresponding RS pulses in 9 cases (group X) and larger in 8 cases (group Y). The Météorage information about the peak currents of the RS pulses and their locations was available for all RS events from the group X but only 2 RS events from the group Y were strong enough to be detected by Météorage. The ratios of the peak-to-peak magnetic field amplitudes of the largest PB pulse and the corresponding RS pulse vary from 0.1 to 0.8 and from 1.4 to 15 for groups X and Y, respectively. The time separation of the first PB pulse and the corresponding RS pulse varies from 0.7 to 46 ms and from 6 to 79 ms for groups X and Y, respectively.

For these 17 sequences followed by the RS, the SAETTA LMA was able to localize VHF radiation sources occurring during the pre-stroke phase in 12 cases. In 5 remaining cases no geo-located VHF radiation source has been found during the PB-RS time period. The number of geo-located sources varies from 0 to 33 in the pre-stroke phase of individual discharges. For the 20 isolated breakdown cases the geo-located VHF radiation sources are always obtained and continue to occur up to the end of our 208-ms long records, their total number varying from 56 to 323 in the individual cases.

In 12 out of 37 cases, the first geo-located VHF radiation source occurs within a 1-ms window centered on the first detectable PB pulse. In the remaining cases the first geo-located VHF radiation source is delayed after the first PB pulse for up to 4 ms in the PB sequences followed by a RS and for up to 13 ms in the isolated breakdown cases. The height above the sea level of all available geo-located VHF radiation sources occurring close to the first PB pulse varies from 1196 to 4474 m. Similarly as in 2012, only a few geo-located VHF radiation sources have been found to be clearly related to the PB pulses and none of the 23 PB pulses detected by Météorage was found among the VHF radiation sources geo-located by the LMA.

**Figure 3:**
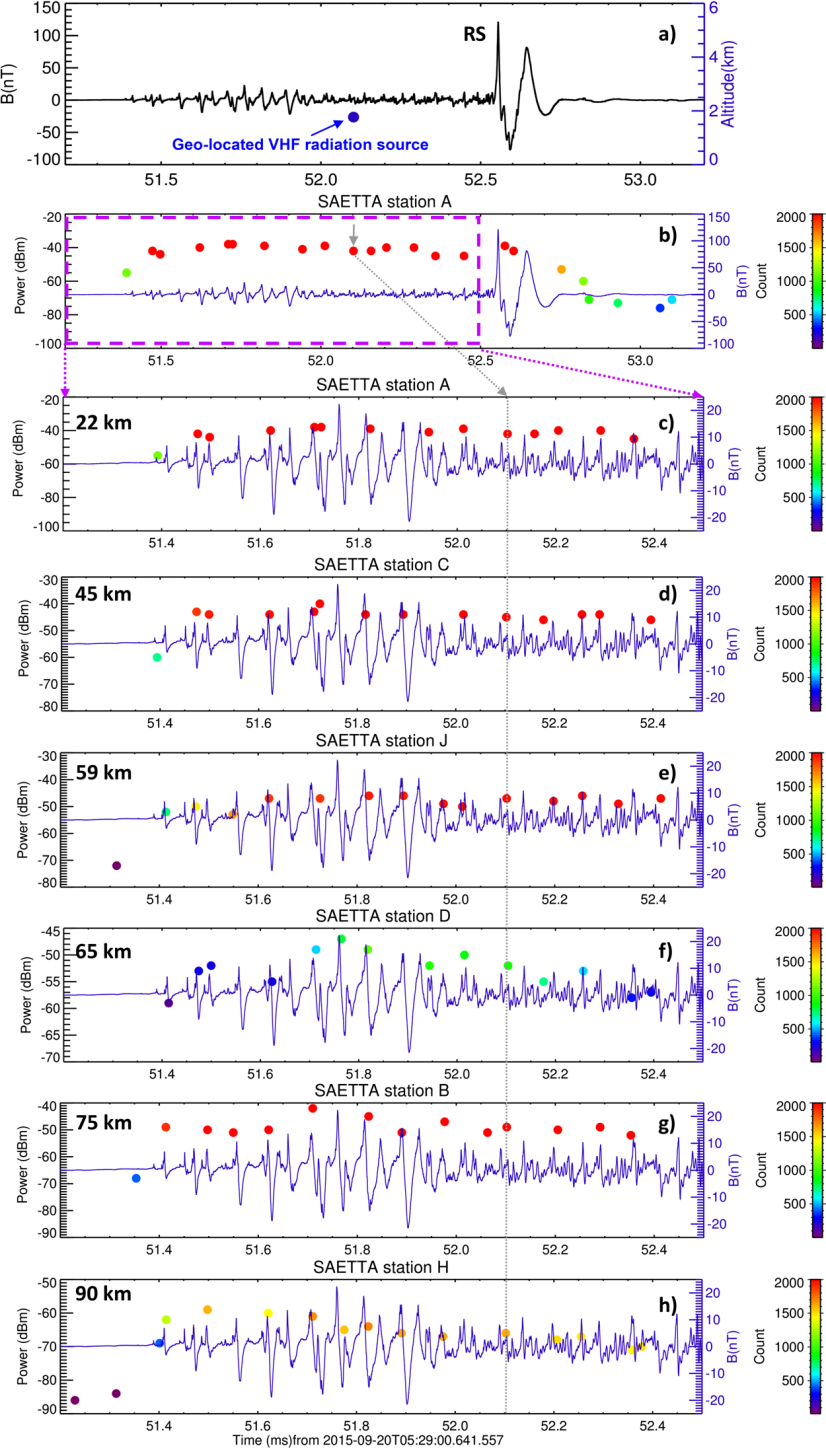
Example of a negative cloud-to-ground discharge from the 2015 dataset. A continuous BLESKA waveform showing a sequence of PBPs and the corresponding return stroke pulse (20 September 2015, 05:29:00.7 UTC, 9.45°E, 42.80°N) from the group X (see text) with (**a**), geo-located VHF radiation sources including their occurence altitudes from the SAETTA LMA and (**b**), peaks of radiated VHF power recorded at the closest SAETTA station A. The abscissas and color-coding are the same as in Fig. 2. (**c–h**) 1.3 ms-long detailed plots showing the PB pulse sequence with the peaks of radiated VHF power detected at different stations (A, C, J, D, B, and H). Gray arrow and line show the only one geo-located VHF radiation source occurring within the pre-stroke period of the observed discharge.

An example of a broadband magnetic-field waveform showing the whole sequence leading to the RS is plotted in Fig. 3a. The data have been recorded on 20 September 2015 at 05:29:00.7 UTC. The RS location reported by Météorage was at 9.45°E, 42.80°N. Its peak current reached −148 kA. Raw records of LMA stations A, B, C, D, H, and J (shown on Fig. 1d) were investigated using the same technique as described for the 2012 dataset. For these LMA stations, Fig. 3b–h show the detected peaks of radiated VHF power together with the broadband magnetic field waveforms. The number of intense VHF samples (above the threshold) per 80-µs time window again reached high values suggesting a continuous VHF radiation. The position of the only geo-located VHF radiation source (marked by a gray arrow) is very close (430 m) to the Météorage RS position.

**Figure 4:**
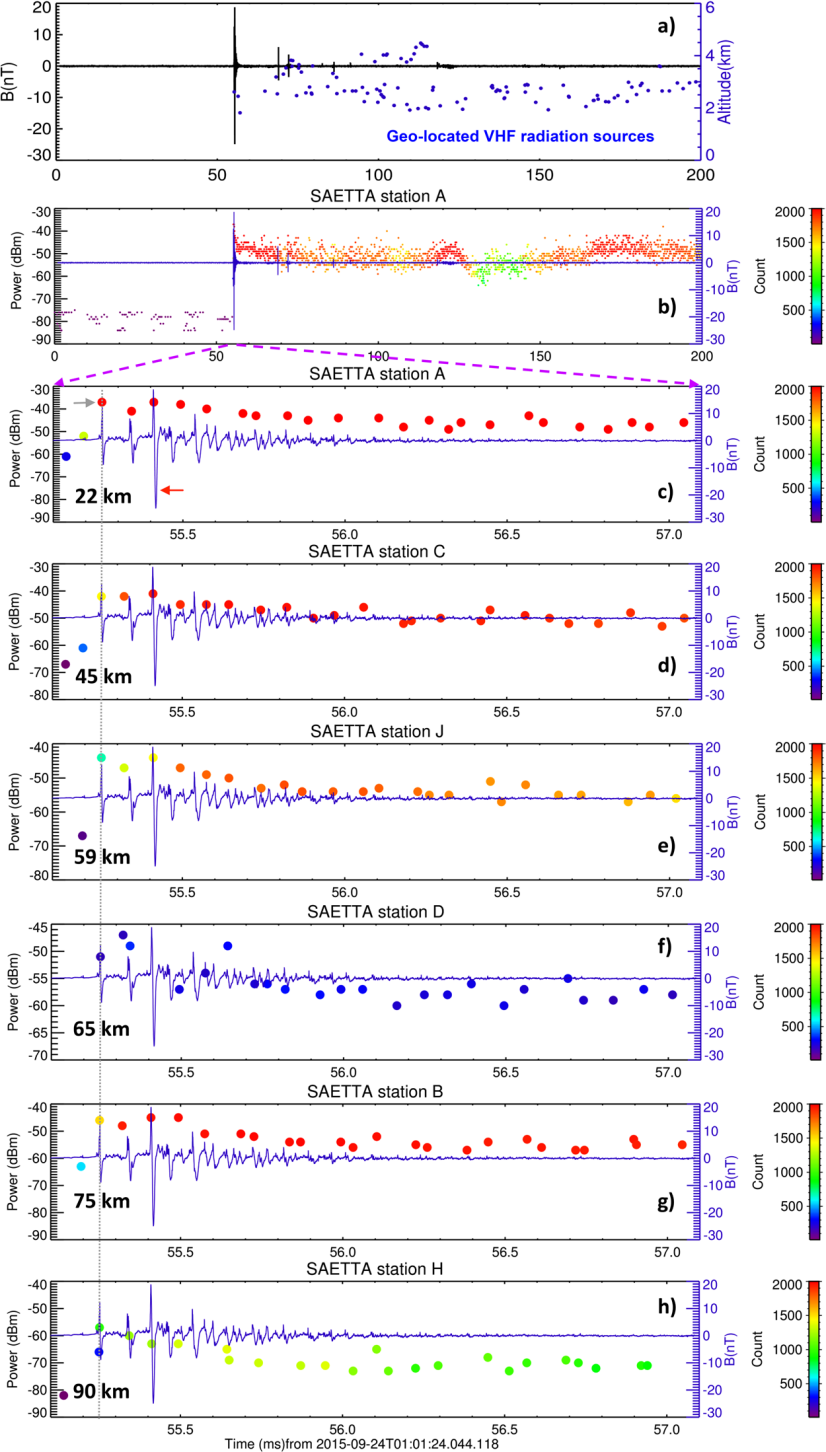
Example of an isolated breakdown event from the 2015 dataset. The same as in Fig. 3 but for a sequence of PBPs and VHF intra-cloud activity on 24 September 2015, 01:01:24.0 UTC, 9.45°E, 42.80°N. Red arrow shows the largest pulse in this sequence which has also been detected by Météorage.

Figure 4a–h show an example of an isolated breakdown process recorded on 24 September 2015 at 01:01:24.0 UTC. Météorage detected the strongest pulse within the PB sequence at location 9.45°E, 42.80°N. The estimated peak current reached 20 kA. Figure 4a and b confirm the continuous intra-cloud activity occurring during the whole 208-ms long magnetic field record. Figure 4c–h show in detail the broadband waveforms of PB pulses in the very beginning of the sequence together with the peaks of radiated VHF power detected at separate LMA stations, giving similar results as the preceding examples. The only geo-located VHF radiation source corresponds to the first larger PB pulse in the sequence (gray arrow). It is found 300 m apart from the above mentioned Météorage location of the strongest PB pulse in the sequence (red arrow). This distance is well below the median value of the position error for intracloud pulses (1.5 km) reported for the National Lightning Detection Network^27^ which is composed of the same type of sensors as those used by Météorage.

## Discussion

Our analysis shows that, using the raw data from separate LMA stations, and taking into account the propagation correction, we find that intense PB pulses and the strongest peaks of VHF radiation recorded at closest stations occur at the same time. Taking into account the technical limitation of our LMA which only detects a single strongest peak of radiated VHF power in each predefined 80-µs LMA window, we obtain nearly one-to-one correspondence of the two processes. This means that the observed peaks of VHF radiated power do not represent a separate phenomenon but rather a manifestation of the same lightning initiation process as the PB pulses.

However, only a few VHF radiation sources which are geo-located by the LMA could be clearly assigned to individual magnetic field PB pulses recorded by our broad-band receiver. In several analyzed cases we have not found any geo-located VHF radiation source occurring within the duration of the PB pulse sequence. This result is in agreement with previous observations^4,24,25,28^, and seemingly confirms the hypothesis that current-producing processes during the lightning initiation which lead to the occurrence of PB pulses are only very weakly related to the sources of impulsive VHF radiation.

It is worth to note here that intense VHF radiation in the LMA data, reflected by high numbers of intense VHF samples, starts synchronously with the first PB pulse. This confirms the presence of VHF radiation at the same time as PB pulses but it also pinpoints the difficulties to reconstruct an accurate geo-location of the VHF radiation sources during continuous radiation processes within the 80-µs LMA time window. Note also that in the 2012 data, only 6 LMA stations were operating during the analyzed period and that for the 2015 data, the LMA stations were deployed in a mountainous environment which influenced the propagation of VHF signals generated by incloud lightning processes.

We have found that the counts of intense VHF samples in 80-µs windows detected at different stations and their received power were quickly decreasing with increasing distance from their source. We also notice that the attenuation of the travelling VHF signals was strongly dependent of the conductivity of the surface^29^. The VHF signals propagated above the poorly conductive mountainous center of the Corsica island (station D, Figs 3f and 4f) were significantly weaker when arriving to the receiving station than signals traveling over similar distances but above the conductive seawater (station B, Figs 3g and 4g).

The high counts of intense VHF samples suggest the presence of a rather continuous VHF radiation in each of the 80-µs LMA windows during the lightning initiation phase. Different stations therefore detected many different peaks, which are only sometimes found by the processing algorithm to correspond to each other. It explains why there were so few geo-located sources within the duration of the PB process. Similar difficulties might have also been at the origin of the previously reported^4,24,25,28^ lack of coincidence of the geo-located VHF radiation sources and individual PB pulses. We therefore think that the conclusions based on previous observations showing that the electric field pulses measured during the initial breakdown phase of intracloud and cloud-to-ground flashes are usually not associated with the geo-located VHF radiation sources might be influenced by the geo-locating performance of lightning mapping arrays.

In summary, results of our analysis of two different datasets confirm that there is a nearly continuous VHF radiation present during the initiation phase of lightning discharges. This situation, in our case also accompanied by a low number of operational LMA stations in one of the datasets, and by a mountainous environment in the other dataset, leads to the lack of geo-located VHF radiation sources and might suggest a conclusion that VHF radiation sources and PB pulses are not related. However, our observations clearly show that the strongest peaks of radiated VHF power received by individual LMA stations are emitted at the same time as intense PB pulses. We think that this coincidence provides us with an electromagnetic evidence of a fast step-like extension of in-cloud lightning channels. These impulsive currents generate impulsive electromagnetic radiation in a wide range of frequencies which can be recorded by a broadband receiver in the form of PB pulses, as well as by individual narrow-band LMA stations in the form of intense peaks of radiated VHF power.

## Methods

### Instrumentation

#### Magnetic field measurement

To detect fluctuations of the horizontal component of magnetic field we use a ground-based version of a broadband (5 kHz–37 MHz) analyzer which was developed for the TARANIS (Tool for the Analysis of Radiation from lightning and Sprites) spacecraft^30^. The sampling rate was 80 MHz and the absolute time was obtained from a GPS receiver with an accuracy of 1 μs. The duration of the waveform snapshots varied from 34 to 208 ms according to the setup of the receiver. The receiver was directly connected to a simple circular magnetic loop antenna^31^ with an effective surface of 0.8 m^2^ and in 2012 it was operated at an external measurement site of the Laboratoire Souterrain à Bas Bruit (LSBB) on the summit of La Grande Montagne (1028 m, 43.94°N, 5.48°E). This device recorded the first group of the measurements analyzed here. The level of the instrumental noise and external interferences allow us to detect broadband pulses exhibiting peak-to-peak amplitudes larger than 1 nT. In 2015, another identical receiver was installed close to Ersa (550 m, 42.97°N, 9.38°E) where the second data set was measured. This time an improved version of a shielded magnetic loop antenna with a versatile integrated amplifier (SLAVIA) was deployed, with a decreased experimental threshold of 0.4 nT.

#### HyMeX and SAETTA Lightning Mapping Arrays

GPS-based three-dimensional LMA systems^19,20^ are widely used narrow-band VHF instruments for tracking the processes occurring inside thunderclouds during the development of lightning flashes. The LMA system measures the time of arrival of impulsive VHF radiation with more than six stations equipped with deployable electric-field antennas and spaced typically 20–50 km apart. Each station detects the peak intensity of VHF radiation in the 6 MHz bandwidth centered at 63 MHz. The sampling rate is 25 MHz^19^.

The arrival time of the strongest peak of radiated VHF power detected by an individual station in each 80-μs time interval (a nominal LMA window) is identified and stored locally on its hard disk. The 3D location of the VHF radiation sources is then calculated using the arrival times of the radiation peaks detected at individual LMA stations. The calculation is based on Euclidian geometry with straight lines of sight. The peaks of radiated VHF power are captured only if their peak amplitude exceeds a threshold which is adjusted individually for each station based on its background noise level^20^. The LMA is able to geo-locate VHF radiation sources also outside of the array up to a distance of about 300 km from the array center.

We have used the data recorded by (1) the HyMeX (Hydrology cycle in the Mediterranean Experiment) LMA operated during HyMeX Special Observation Period 1 (Sept to Nov 2012)^32^ and (2) the SAETTA (Suivi de l’Activité Electrique Tridimensionnelle Totale de l’Atmosphère) network operated in Corsica since June 2014. The HyLMA (HyMeX Lightning Mapping Array) was composed of 12 stations. It was deployed in South-West of France in the area of the Mediterranean seaside. The SAETTA lightning mapping array is also composed of 12 LMA stations that are spread over Corsica. Both LMA systems were set to achieve reliable geo-location of VHF radiation sources, when at least six stations detect the same source. GPS receivers connected to each LMA station provide accurate time assignment.

#### The Météorage operational network

Locations, polarities, and peak currents for a subset of observed discharges were provided by the French lightning locating system (LLS) Météorage. It is composed of 21 sensors LS7002 installed across France. In addition, to the French sensors, the system combines the measurements of sensors operated by Italian national LLS (SIRF) which ensure an optimum coverage of the South-East France and Corsica regions. The major evolutions of the system between 2012 and 2015, the two periods of considered in this study, consisted of an enhancement in the sensors sensitivity and an upgrade of the electronics leading to a better signal processing. After this upgrade, the detection efficiencies for strokes were evaluated to 94%, the median location accuracy was estimated to 120 meters^33^ and the accuracy of estimation of peak current amplitudes is about 18%^34^. The procedure**s** for estimation of peak currents for cloud-to-ground and intracloud lightning are identical. Only cloud-to-ground discharges were included in the Météorage list in 2012. The characteristics of both cloud-to-ground and intracloud discharges were available in 2015.

### Data availability

The data used in this study are available at http://bleska.ufa.cas.cz/.
